# Navigable rivers facilitated the spread and recurrence of plague in pre-industrial Europe

**DOI:** 10.1038/srep34867

**Published:** 2016-10-10

**Authors:** Ricci P. H. Yue, Harry F. Lee, Connor Y. H. Wu

**Affiliations:** 1 Department of Geography, The University of Hong Kong, Hong Kong; 2 International Center for China Development Studies, The University of Hong Kong, Hong Kong; 3 Department of Population Health Sciences, Virginia-Maryland College of Veterinary Medicine, Virginia Tech, US.

## Abstract

Infectious diseases have become a rising challenge to mankind in a globalizing world. Yet, little is known about the inland transmission of infectious diseases in history. In this study, we based on the spatio-temporal information of 5559 plague (*Yersinia pestis*) outbreaks in Europe and its neighboring regions in AD1347–1760 to statistically examine the connection between navigable rivers and plague outbreak. Our results showed that 95.5% of plague happened within 10 km proximity of navigable rivers. Besides, the count of plague outbreak was positively correlated with the width of river and negatively correlated with the distance between city and river. This association remained robust in different regression model specifications. An increase of 100 m in the width of river and a shortening of 1 km distance between city and river resulted in 9 and 0.96 more plague outbreaks in our study period, respectively. Such relationship shows a declining trend over our study period due to the expansion of city and technological advancement in overland transportation. This study elucidates the key role of navigable river in the dissemination of plague in historical Europe.

In a globalizing world, infectious diseases have become a rising challenge to mankind^1^,^2^,^3^,^4^. The Zika virus infection in central and South America in 2016, the avian flu outbreak in Korea in 2015 and Ebola outbreak in West Africa in 2014 remind us that when the world is interconnected in all possible fronts of mankind, including flow of population, trade, influx of information and exchange of culture and capital, regional infectious disease outbreak could be quickly ended up as global crisis^5^. The situation has been getting worse as the amplified circulation of bioactive nitrogen compound and rapid loss of habitat has apparently pushed this planet to an ‘unsafe’ level^6^. Nevertheless, little is known about the transmission of infectious diseases across different spatial domains in history^7^. Particularly, the way of how different environmental and socio-economic factors mediate the spatial spread of infectious diseases is under-researched, probably owing to scarce historic big data for scientific analysis.

In this study, we focused on the spatial spread of plague in relation to geographic factors in recent history, which may serve as future capital for controlling infectious diseases. According to World Health Organization, thousands of plague cases were still recorded almost every year in previous decades^8^,^9^. Except for extensive research in medical discipline, existing studies of plague emphasize on the identification of natural foci, distribution of plague location in different periods, influence of climate change on plague outbreak and exact origins of vector of plague outbreak. Although there are several studies on the spatial spreading mechanism of plague, the exact pathway (particularly for inland transmission) still remains elusive. Xu, *et al.*^7^ suggest that the presence of major roads, rivers and coastlines shaped and speeded up the transmission of plague in historic China. On the other hand, Enscore, *et al.*^10^ and Parmenter, *et al.*^11^ highlight the association between climatic variables and plague outbreak in USA. This conclusion is also drawn in the empirical studies in Kazakhstan^12^ and Madagascar^13^. Several environmental and socio-economic parameters are envisaged to be significant determinants of plague dynamics.

Here we analyzed plague outbreak across the European continent and northern Africa in AD1347–1760. Black Death (AD1347–1353) was the greatest epidemics in human history, leading to the death of half of the population in Europe over its outbreak^14^. A recent DNA research conducted by Haensch, *et al.*^15^ identifies *Yersinia pestis* as the cause of plague in Europe during the Black Death and the four centuries afterwards. Such a history provides a substantial number of plague outbreak incidents over various spatio-temporal domains for analysis. Even though the plague dynamics in Asia, Africa and North America are relatively well understood, the one in pre-industrial Europe remains insufficiently explored.

We focused on the plague outbreak that happened in the inland of European continent. Schmid, *et al.*^16^ recently conclude that wildlife reservoir for *Yersinia pestis* never existed in medieval Europe. The source of all plague outbreaks was originated from the imports through Silk Road, either by ships or by major trade routes. Theoretically, if cases that are transmitted through maritime trade routes are discounted, every inland plague outbreak incident is caused by previous plague outbreak nearby. Our study period mainly lies before the technological transformation of transportation when motorized transportation was only brought forward at the end of the 18^th^ century^17^. People relied heavily on water transport for both trade and transportation. During the time of Medieval Europe, the cost of water transport was at least 10 times cheaper than that of land transport^18^. Besides, navigable rivers also linked up cities further apart from each other. Animal labors provided basic land transportation means among adjacent settlements, while river played a key role in connecting distant cities when people were not willing to spend weeks travelling on roads.

We hypothesized that trade and transportation brought by navigable rivers was an important medium for the spread of plague in European inland. To prove this hypothesis, we extracted historic plague records from Büntgen, *et al.*^19^ geo-referenced plague database. OLS regression estimates were employed to investigate the influence of navigable rivers on plague outbreak. Different sets of geographic and socio-economic factors were entered into the regression models, with their individual and combined effect on plague outbreak controlled/estimated. We then based on the resultant coefficients of association to determine the contribution of navigable rivers on plague outbreak.

Further we aggregated our data according to the number of plague outbreak and took the averaged width of river and averaged distance between city and river as parameters to estimate the association between navigable rivers and plague outbreak. We then suggested the possible pathways of how rivers determined the pattern of plague outbreak in pre-industrial Europe. We sought to shed light on inland transmission of plague in pre-industrial Europe and hence, contributing to plague prevention in underdeveloped world nowadays.

## Results

### General relationship between rivers and plague outbreak

**Figure 1:**
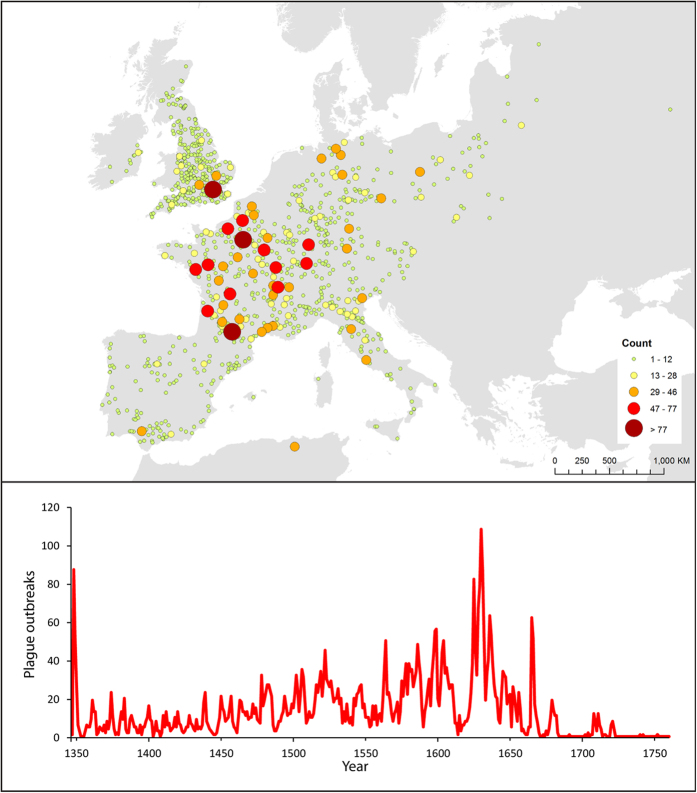
Temporal and spatial distribution of plague outbreak in Europe in AD1347–1760 (modified from Büntgen 2012). Plague incidents that happened within 5 km from the coastline were excluded. The frequency of plague outbreak is divided by fixed intervals and the size of dot represents the frequency of plague reoccurrence. The study area includes the major continent of Europe (exclude Northern Europe), British Isles and small fraction of Northern Africa. The map is generated in ArcGIS version 10.1 ( www.esri.com/software/arcgis).

Our study period spanned between the onset of Black Death (AD1347) and the start of Industrial Revolution (AD1760) (Fig. 1). Plague was most dominant during Black Death (AD1346–1353) and the Thirty Years’ War (AD1618–1648)^19^. Of the 5559 plague outbreaks in our study period, 95.5% of them happened within the 10 km proximity of navigable rivers (Table 1). Also in terms of the proportion of cities with plague outbreak against all cities (Table S1), plague reoccurrence declines against increasing distance intervals. Among those cases, 84.0% of them were found in cities, with their city centers only 1 km diameter away from rivers. Out of those rivers, 79.5% of them are ≥20 m in width, while the average width of river is 84.6 m (Table S2). Before Industrial Revolution, European cities were rarely larger than 2.5 km in radius^17^. The above figures reveal that most plague outbreaks happened in cities with a navigable river just next door or running through. Yet, it is reminded that such association is not conclusive as we do not contain the relative distance of rivers and their width in all historical Europe cities. To reveal the real association between navigable rivers and plague outbreak, we applied OLS estimates on the count of plague outbreak, width of river, closest distance between city and navigable river and other potential variables.

Results of our OLS regression estimates are summarized in Table 2. Column (1) depicts the influence of the width of river and city-river distance upon the number of plague outbreak, with time fixed- and region fixed-effects applied. Both factors are statistically significant (*p* < 0.01) in explaining the variation of number of plagues happened across Europe, with a R^2^ value of 35%. As reported, the 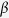 value for the width of river and for the city-river distance is positive and negative, respectively. The wider and closer a river was in a city, the easier the plagues would be transmitted to there.

In column (2), we included spatial lag in the regression models. Here spatial lag represents the existence of spatial dependency of plague outbreak points nearby (see SI Text and Fig. S2). In any model followed, the number of plague outbreak remains highly significantly associated with the spatial lag (*p* < 0.01), indicating that plague outbreaks were spatially dependent to previous outbreaks in adjacent cities. Despite the inclusion of the spatial lag, the significance and direction of the coefficients of the width of river and the city-river distance were largely unaffected.

In column (3), we considered the effect of geographical factors on plague outbreaks. These factors basically capture the climatic condition of the plague outbreak locations. Our results indicate that Western Europe was more prone to the reoccurrence of plague outbreak. Besides, highland regions were less favorable to plague outbreak. It could be attributable to the lack of navigable rivers in highland. Out of the 5559 plague outbreaks in our study period, only 20 of them occurred 1000 m above the sea level (a.s.l.) (Fig. S3).

In column (4), we further controlled those variables pertinent to the change of urban environment across space and time. The degree of vegetation cover in usable land reveals urban development over time. We also included normalized population density to show whether population in city would affect the river-plague relationship. In column (5), we incorporated control variables that capture the regional variation of economic status among different plague outbreak locations, such as GDP per capita, consumer price index and normal wage of labors. Yet, none of the above control variables were statistically correlated to the count of plague outbreaks, implying that plague spread regardless of the regional variation of population density and economic status in pre-industrial Europe. Besides, the inclusion of those control variables did not affect the statistically-significant association between plague outbreak and the width of river/city-river distance.

Within the OLS estimates, we also obtained the standardized beta coefficient 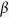, which shows that contribution of each factor to the risk of plague reoccurrence. With the large-N (n = 5559) data, we are interested in estimating the effect of navigable rivers on plague outbreak within our study time-span. The standardized beta coefficients of the width of river and the city-river distance differ across columns (1) to (5). For the width of river, it ranges from 0.32 to 0.35. For the city-river distance, it ranges from −0.08 to −0.10. According to column (5) of our OLS estimates, in a controlled setting, an increase of 100 m in the width of river would yield nine more plague outbreaks in a city. Besides, a shortening of 1 km city-river distance would increase the count of plague outbreak by 0.96 unit (Fig. S4).

### Stationarity and aggregation of the river-plague relationship

The OLS estimates provide quantitative evidence that the width of river and the city-river distance were associated with the number of plague outbreak in pre-industrial European cities. Subject to the expansion of city and advancement of land transportation, we hypothesized that the importance of inland waterway to plague outbreak would remain statistically significant but less distinct over time.

We preformed various sensitivity tests to examine the stationarity of the river-plague relationship in different time sections. Results are reported in the first half of Table 3. Both of the variables were statistically significant. The coefficient for the width of river remains positive over time, suggesting that the wider the river the greater the chance plague recurred in a city. Besides, the coefficient for the city-river distance stays negative over time, which means a river closer to city would increase the chance of plague recurrence. Nevertheless, when comparing the R^2^ generated in each model, we observed that the explanatory power of the width of river and the city-river distance weakened over time. In AD1347–1449, R^2^ was 50%, indicating that the equivalent percentage of variation in total number of plague outbreak can be explained by these two variables. In AD1450–1549, R^2^ dropped to 38%. The R^2^ decreased to 31% when we analyzed the plague outbreak in the next 100 years. Finally, in AD1650–1760, R^2^ fell to 27%, which is almost half the amount of that in AD1347–1449. However, given our big sample in each time section, the explanatory power is still considered high and significant in elucidating the frequency variation of plague outbreak.

In the second half of Table 3, we also evaluated the robustness of our sample with OLS estimates after data aggregation, with cities having the same number of plague outbreak aggregated together as analytic units. The result in check (E) suggests that both the width of river and the city-river distance were significantly correlated with count of plague outbreak. In check (F) we manually removed samples from Russia and Turkey – locations that are too far away from the main European continent. In check (G) we only aggregated data from UK, France, Germany and Italy since they shared 81.9% of plague outbreaks in our dataset. Both columns show a consistent and robust relationship between plague occurrence and the dimension and location of river crossing the city.

**Figure 2:**
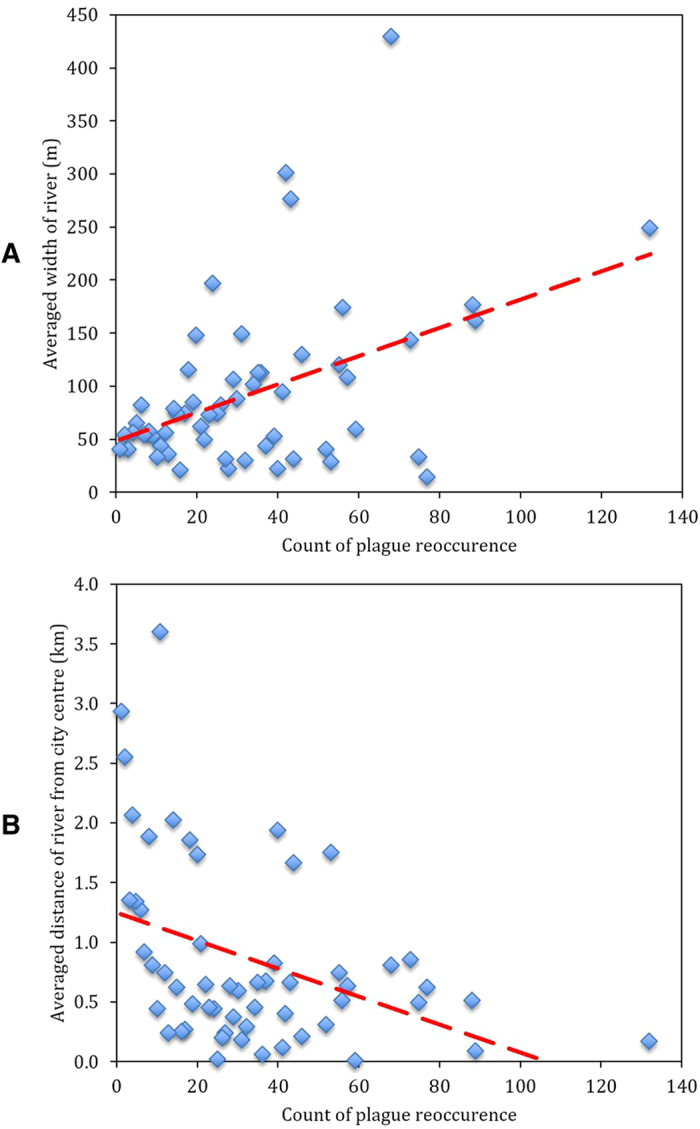
Relationship between navigable river and plague outbreak in Europe, AD1347–1760. (**A**) Averaged width of river and plague outbreak. (**B**) Averaged city-river distance and plague outbreak. The dashed line represents the trend line.

Figure 2 shows the relationship between river and number of plague outbreak when all data were aggregated according to the count of plague outbreak. We took this as a further step to verify the association we were looking into. By aggregating data under the same number of plague outbreak, we could show how the river-plague relationship goes in a more macro-perspective. There is a clear trend that the width of river holds a positive association with the total number of plague outbreak, while the city-river distance holds a negative association with the plague outbreak.

Our results confirm that the strength of the river-plague outbreak relationship weakened over time, probably due to the technological advancement of land transportation and the expansion of city. The results correspond to our hypothesis that indicates the causal relationship between navigable rivers and plague outbreak. Also, even if the relationship is examined in various spatio-temporal combinations, it still remains robust and statistically significant.

## Discussion

Before the invention of railways, water transportation was imperative for trade and exchange, linking up population and city together^20^. Substantial amount of literature has indicated maritime trade routes as a crucial factor in the metastatic transmission of infectious diseases across countries and continents^16^,^21^,^22^. We further drilled into the question of how far the spread of plague was determined by geographic factors in pre-industrial Europe. Our statistical results showed that while people and goods (especially grains) were carried along inland rivers, so did plague. *Yersinia pestis* first ridded on rodent in its flea hosts, and these rodents followed the grains which would usually be transferred up and down the rivers, thus encouraging the spread of *Yersinia Pestis* to larger and border communities. An important consequence of this spreading pattern was that plague tended to spread to cities where trade was heavily dependent on rivers. Hence, 95.5% of the plague cities contained a navigable river. Land transportation might be a less likely means to transmit the disease between two separate epi-centers as it led to the dehydration of goods, which is unfavorable to the fleas. Alternatively, the humidity given by voyage provided essential support for the fleas to survive for weeks^14^. Besides, infected rat could survive for a few days, while infected humans would die in 14.5 hours on average^23^. Hence, it was the trading of goods (which could contain infected rat), rather than the transportation of humans, facilitated the spread of plague. The above synthesis also implies that city with more frequent river trading would have a higher likelihood of plague outbreak. Our OLS estimates proved this hypothesis. Konings^24^ shows the width and the distance of river to be the indicators of transportation volume of inland waterway. A wider river not only allowed larger ships to pass through, but also allowed more facilities to be built to enhance trading. A distant river, however, increased physical and time constraints on trade and transportation^17^ and hence, reduced their volume. Our results could evidence the historic observation about the connection between trade volume and the outbreak/recurrence of plague in a city, which was mediated by the width of river and the distance between city and river.

We are aware that flooding event and drought events could have a plausible impact to plague outbreak. A number of studies have pointed out that flooding, drought or higher/lower than normal precipitation would stimulate change in rodent ecology^11^,^25^,^26^,^27^. In these circumstances, rodents would be driven out to compete for food with humans in urban area, causing more interactions between the rodent fleas and humans. These factors again favour cities around river to become plague outbreak points. Yet, it has been emphasized that the width of rivers (i.e., our criterion in defining rivers) does not necessarily correlate with the frequency of extreme hydrological events^28^. Therefore, the above mechanism is not contradictory with our conclusion that trading at rivers primarily controls the spatial distribution of plague outbreak.

Also, we found the strength of river-plague relationship to be weakened over time. After the Black Death, Europe came back quickly in both the economy and population during the Renaissance. In AD1400–1750, the population in Europe doubled^29^, implying that urban area had to expand to cater for the growing population. Such phenomenon might have distorted the effect of the city-river distance on plague. Generally, population density was proportional to prosperity in pre-industrial era^30^. The more the people, the more the resources could be invested on technological advancement in societies. The influence of technological improvement was two-fold. First, it helped to reduce the barriers to navigate in small rivers and also shorten the circulation time between water and land. Second, technological advancement helped to switch waterway transportation to land transportation. Comparing to the late medieval time when land transportation was ten times more expensive than waterway transportation^18^, the discrepancy dropped to about two to four times in early modern age^31^. The increasingly cheaper land transportation might have further weakened the river-plague relationship in pre-industrial Europe over time.

Another explanation to the weakened river-plague relationship over time could be attributed to the active immunization of humans to plague. Yet, despite of the early support by Hirst^32^ and McNeill^33^, the hypothesis has not been generally accepted in the academia^34^,^35^. Owing to the lack of evidence to show that bubonic *Yersinia pestis* reduce its damage to humans over time instead of mutating to a more lethal one. Besides, the hypothesis faces the fundamental critique that mortality rate remained high during the third pandemic (AD1855–1959) when the immunization was available.

While plagues in Europe were spread via inland waterways, their outbreak was also influenced by other geographic factors associated with the waterways. Elevation is highlighted as one of those factors, as upper streams are usually narrower and more difficult for water transportation, so does the spread of plague. An example from medieval England might evidence this phenomenon: At the end of medieval Europe, the inland waterways at upstream were rarely navigable due to the increasing set up of obstructions, silting and government policy^18^. In our dataset, the highest point of plague outbreak in England was from a city at only 299 m a.s.l., while the average elevation of plague outbreak in England was 47.5 m a.s.l.

The situation in Rivers Derwent, Greatriver Ouse and Fossdyke were especially serious when these waterways were severely silted and blocked for navigation after the 14^th^ century^18^ (Fig. S5). Although plague outbreak seemed to cover the entire UK, we could find that number of plague outbreak diminished along the blocked part of major waterways in UK. In River Derwent, plague outbreak focused at the two ends of the blocked canal, which are further connected to major canal system of UK. However, within the blocked parts, there were only two plague cases being notified. In the case of Fossdyke, plague stopped spreading upward from York although York was one of the major plague hotspot in UK. In the case of Greatriver Ouse, we find only one case of sporadic plague outbreak, while plague cases were spotted frequently in other part of Greatriver Ouse. From these historical evidence, plague seems to have difficulties spreading to the blocking part of these rivers/canals. It might be rather preliminary to conclude that the blockage of those rivers drove plague away from inland waterways. There is a lack of available literature to validate such hypothesis in medieval and early modern Europe. Yet, such possibility should not be disregarded. We recommend the academia to further testify this hypothesis in a more recent database (i.e. third pandemic in China) or other local scale study.

There are limitations in this study. Our results are based on the database compiled by Büntgen *et al.*^19^. However, no database could include all real cases in history. It is at best a recovery of history from places with less damaged records. Also, our measurements of river derived from present day’s data. Some rivers might have been modified by humans (i.e., straightened, reclaimed, widened, or even directed to underground tunnels). Finally, our statistical models offered a generalized explanation to the river-plague connection at the macro-scale, while contextual factors such as social responses and policy measures during plague outbreak could determine the spread of plague at the micro-scale.

Our results enable a deeper understanding of inland plague transmission. We statically evidenced that the presence of navigable rivers significantly altered the spreading pattern and recurrence of plague in pre-industrial Europe. A wider and closer river to city was associated with more trade and transportation, which could facilitate the spread of plague. On the other hand, this relationship was weakened by the advancement of transportation and expansion of city size. Nevertheless, the river-plague relationship remains highly significant throughout the study period, which is backed up by historic observation.

## Materials and Methods

### Data

Our plague data came from the digitalized plague database compiled by Büntgen, *et al.*^19^. We extracted plague outbreak record in AD1347–1760 according to the onset time of outbreak. To isolate the influence of maritime trade routes from that of navigable rivers, plague incidents that happened within 5 km from the coastline were excluded. There are 5559 plague outbreaks from 1001 inland cities in total. Since the dataset does not distinguish the exact onset date of plague outbreak, we treat multiple plague outbreaks in any given city in the same year as a single plague outbreak incident. For the river-related data, ‘width of river’ was measured in meter. Nowadays, the vessels would only be permitted to navigate at river of about 8 m in width^36^. However, considering the size of ancient boats that were used for waterway navigation^37^ and navigation history in small river in central Europe^38^, we took 5 m as the minimum width to define navigable rivers. To ensure those identified water body could serve as trade routes, there are two more requirements: (1) those river should be connected with other cities; and (2) enclosed lake without navigable river flowing to other cities is neglected. The digitalized dataset from Büntgen, *et al.*^19^ provides the latitude and longitude information of those cities with plague outbreak. Based on that information, together with the locational information of navigable rivers, we used ArcGIS to measure the ‘city-river distance’. If no river is found within the 10 km radius of a given city, 0 m and 10000 m will be manually set as the width of river and the city-river distance for that city, respectively.

### Control variables

There was never a natural reservoir of plague focus in historical Europe^16^, implying that the plagues there were transmitted from elsewhere. This might have resulted in the spatial clustering of events and consequently biased the association we focused on^39^. Therefore, we applied ‘spatial lag’ to control the cross-sectional correlation of the condition and involvement of nearby unit to the likelihood of plague outbreak of any unit^40^. Besides, we also applied geographic and socio-economic controls in our statistical models. For the geographic control variables, we took ‘distance from the Equator’ as the value of latitude difference between a given city and the Equator. The value of ‘longitude’ came from the plague dataset of Büntgen, *et al.*^19^. Both of them are measured in degree. ‘Elevation’ is measured by ArcGIS according to the coordinates of each plague outbreak point. For the socio-economic control variables, percentage of usable land across historical Europe was calculated according to the ‘vegetation cover’ database of Kaplan, *et al.*^41^. ‘Normalized population density’ was generated by combining the datasets of Kaplan, *et al.*^41^ and McEvedy and Jones^29^. ‘Per capita GDP’ came from Bolt and Zanden^42^ and Maddison^43^. ‘Consumer price index’ and ‘normal wage’ were obtained from Allen^44^.

### Statistical analysis

We employed OLS estimates to examine the association between the counts of plague outbreak and the width of river/city-river distance. We hypothesized that the presence of navigable rivers significantly altered the spread of plague. When examining this relationship, other variables that might also affect plague outbreak were controlled. Our ‘hypothesized scenario’ is expressed in the following equation:
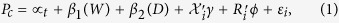
where *P*_*c*_ is the total count of plague outbreak in a city, *α*_*t*_ indicates time fixed-effects. W stands for the width of a city’s closest river, D stands for the distance between a city (its centroid) and its closest river. 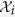 is the vector of other observed explanatory variables we controlled. 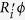 refers to the country fixed-effects for plague outbreak. *ε*_*i*_ is the error term. *β* is the coefficient we are interested in. *β*_1_ is the estimated relationship between the width of river and the number of plague outbreak at a city. *β*_2_ is the estimated effect of the city-river distance on the number of plague outbreak at a city.

## Additional Information

**How to cite this article**: Yue, R. P. H. *et al.* Navigable rivers facilitated the spread and recurrence of plague in pre-industrial Europe. *Sci. Rep.* **6**, 34867; doi: 10.1038/srep34867 (2016).

## Supplementary Material

Supplementary Information

## Figures and Tables

**Table 1:** Cumulative percentage of plague outbreak incidents in relation to navigable rivers in Europe, AD1347–1760.

Radius of measurement (R) (km)	10	7.5	5	4	3	2	1
Plague outbreak with a navigable river within R	5309	5271	5216	5174	5065	4966	4461
Total Plague outbreak	5559	5559	5559	5559	5559	5559	5559
Percentage (%)	95.5	94.8	93.8	93.1	91.1	89.3	80.2

**Table 2:** OLS regression models for the total count of plague outbreak per outbreak point in Europe, AD1347–1760.

	Model 1	Model 2	Model 3	Model 4	Model 5
Width of River	0.0995185*** ( 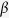 = 0.351109)	0.0962446*** ( 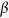 = 0.3395736)	0.0918961*** ( 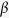 = 0.3242312)	0.0919283*** ( 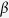 = 0.3243449)	0.0919559*** ( 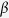 = 0.324442)
Distance of River from city centre	−1.276682*** ( 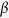 = −0.1008757)	−1.25114*** ( 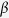 = 0.0988647)	−0.9758854*** ( 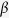 = −0.0771141)	−0.9794637*** ( 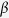 = −0.0772024)	−0.9771168*** ( 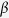 = −0.0772132)
Spatial Lag		−9.275146*** ( 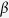 = −0.1334507)	−9.128437*** ( 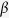 = −0.1313398)	−9.147805*** ( 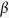 = −0.1316185)	−9.084119*** ( 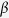 = −0.1307022)
Distance from equator			−0.2338274 ( 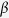 = −0.032463)	−0.2310886 ( 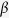 = −0.0320828)	−0.2205209 ( 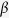 = −0.0306156)
Longitude			1.048405*** ( 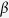 = 0.22237368)	1.049983*** ( 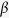 = 0.2227083)	1.041675*** ( 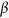 = 0.22094628)
Elevation			−0.0223869*** ( 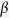 = −0.1387659)	−0.0223048*** ( 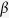 = −0.1382572)	−0.0223221*** ( 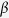 = −0.1383645)
Vegetation Cover				( 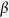 = −0.1649253)	−0.6105889 ( 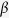 = −0.1635749)
Normalized Population Density				0.000591 ( 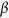 = 0.006708)	( 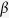 = −0.0197739)
Per Capita GDP					−0.010114 ( 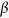 = −0.076168)
Consumer Price Index					−0.2604331 ( 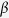 = 0.003831)
Normal Wage					−0.2021959 ( 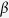 = −0.0097477)
Time Fixed effect	Yes	Yes	Yes	Yes	Yes
Regional Fixed effect	Yes	Yes	Yes	Yes	Yes
Number obs.	5559	5559	5559	5559	5559
R^2^	0.35	0.36	0.37	0.38	0.38

Notes: The dependent variable is total number of plague count in study period.

***p < 0.005.

**p < 0.01.

*p < 0.05.

**Table 3:** Robustness check for the OLS estimates of beta.

Check	Width of River	Distance of River from city centre	Number of obs.	F	R^2^
First part: Sensitivity check across temporal domain					
A. Using only plague outbreak during AD1347–1449					
	0.1096634*** (0.3507051)	−1.053141* (−0.742794)	777	6.00	0.50
B. Using only plague outbreak during AD1450–1549					
	0.0929646*** (0.315163)	−1.525797*** (−0.1034473)	1660	8.15	0.38
C. Using only plague outbreak during AD1550–1649					
	0.1194134*** (0.404504)	−1.267226*** (−0.1084678)	2667	9.84	0.31
D. Using only plague outbreak during AD1650–1760					
	0.0198132** (0.1449958)	−1.186286*** (−0.1735572)	455	2.28	0.27
Second part: Sensitivity check across spatial domain					
E. Aggregating cities with the same number of plague outbreak					
	0.1272769*** (0.3794992)	−10.69387** (−0.3156618)	56	11.10	0.30
F. Dropping all cases in Russia and Turkey before aggregating cities with the same number of plague outbreak					
	0.1266675*** (0.3800006)	−10.6744** (−0.3155398)	56	11.18	0.30
G. Aggregating cities with the same number of plague outbreak only in UK, Germany, France and Italy					
	0.1417548*** (0.424572)	−8.895052* (−0.2789527)	54	11.05	0.30

Notes: The dependent variable of check A, B, C and D is the total number of plague reoccurrence within our study period. Time fixed effect and regional fixed effect were added to the model. For check E, F and G, the dependent variable is number of count of plague outbreak. Width of river and distance of river away from city centre are aggregated and averaged for check E, F and G before statistical analysis. No fixed effect was added to the model.

***p < 0.005; **p < 0.01; *p < 0.05.
