# Supplementary material for: Navigable rivers facilitated the spread and recurrence of plague in pre-industrial Europe
Source: Sci Rep. 2016 Oct 10;6:34867. doi: 10.1038/srep34867 (PMC5056511; doi:10.1038/srep34867)
Supplement: Supplementary Information [file srep34867-s1.pdf]

Navigable rivers facilitated the spread and recurrence of plague in pre-industrial Europe

Ricci P.H. Yue [a*], Harry F. Lee [a, b], Connor Y.H. Wu [c]

[a] Department of Geography, The University of Hong Kong
[b] International Centre for China Development Study, The University of Hong Kong
[c] Department of Population Health Sciences, Virginia-Maryland College of Veterinary Medicine, Virginia Tech

[*] Corresponding author. Tel.: +852 6504 0880; fax: +852 2559 8994.
E-mail address: ricciyue@hku.hk (R.P.H. Yue)

Supplementary text

Table S1. Proportion of plague-infected cities against all cities at different distance interval to navigable rivers.

Table S2. Summary statistics of the variables employed in our OLS estimates.

Figure S1. Spatial distribution of plague outbreak in Europe in different periods. The frequency of plague reoccurrence is corresponding to the colour spectrum. (A). AD1347 – 1449. (B). AD1450 – 1549. (C). AD1550 – 1649. (D). AD1650 – 1760. The maps are generated in ArcGIS version 10.1 (www.esri.com/software/arcgis).

Figure S2. Graphic illustration of spatial lag.

Figure S3. Distribution of elevation of plague outbreak incidences in Europe, AD1347 – 1760.

Figure S4. Theoretical impact of river to plague outbreak in Europe, AD1347 – 1760. (A). Theoretical width of river in relation to relative increase in plague reoccurrence. The standardized beta coefficient from column (5) of Table 2 is used. (B). Theoretical distance away from city centre in relation to relative increase in plague reoccurrence. The standardized beta coefficient was brought from column (5) of Table 2.

Figure S5. Maps showing Rivers Derwent, Greatriver Ouse and Fossdyke and the closest plague outbreak point nearby. The maps are generated in ArcGIS version 10.1 (www.esri.com/software/arcgis).

Supplementary text

Detailed data description. Here we explained our rationale on the selection of study area, study period and calibration of data. For our study area, Europe is picked up since it is one of the areas with the most extensive historical plague outbreak record being digitalized. Part of North Africa is also included as the dataset by Büntgen, et al. [1] documented the plague outbreak within the area. Concerning the availability of other social, demographic and economic attributes, Europe and North Africa are considered as sufficient. Our data focuses mainly on the main continent of Europe without Northern Europe, British isles and major coastal cities of North Africa. The land relief of Europe is complicated. The central highland/mountainous area (i.e., Alps and Pyrenees) are surrounded by descending lowlands. The combination of alternate plateaus and lowlands create a vast system of river basins. The major navigation waterway systems usually start from the highland and flow through the major plain area at low elevation. These waterway systems include Rhine, Thames, Weser, Seine, Minho and so on. Before the invention of railways during the Industrial Revolution era, these navigable rivers serve as the major trade routes connecting different settlements and cities together [2].

For our study period, it starts in AD1347. It is the earliest year that our plague outbreak database by Büntgen, et al. [1] could reach. According to the literature review done by Benedictow [3], AD1347 is also the year that plague was reportedly introduced into Europe. Therefore, AD1347 is picked up as the starting year of our study period. Our study period ends at AD1760, which is regarded as the starting point of Industrial Revolution [4]. Since then, technological breakthrough started to modify the pattern in hygiene, medicine and human movement. Our database also shows a significant decline of plague outbreak after AD1760 (Fig. S1). By focusing at the period before the Industrial Revolution, we can more clearly elucidate the role of inland waterway in shaping the distribution of plague outbreak in historical Europe. The summary statistics of all variables we used in our OLS estimates are provided in Table S1.

Plague dataset. The plague dataset is originated from the work of Biraben [5]. His inventory documented 11180 outbreaks out of literature review from various sources. The inventory was validated by Büntgen, et al. [1]. Uncertainties and imprecise description of data were removed before they transformed the inventory into a digitalized spatiotemporal database. A total of 6929 plague outbreaks in AD1347–1900 were geo-referenced across Europe and North Africa. In our study, we highlighted the plague outbreaks from a period of AD1347–1760 to eliminate the period after the industrial revolution. We also manually remove plague outbreaks locating at a distance less than 5km to the coastline to make sure that we are testing only on inland plague outbreaks. Recurring outbreaks in the same year were considered as single outbreak since the geo-referenced database provided by Büntgen, et al. [1] did not provide the onset time of plague outbreaks. At last a total of 5559 plague outbreaks were included in our analysis.

Width of river and city-river distance. First we use the coordinates provided by Büntgen, et al. [1] as the reference point for each plague outbreak city. These coordinates were entered into ArcGIS v10.1 to find out its closest navigable river. To define 'navigable', we borrow the current standard that vessels would only be permitted to navigate at river of about 8m in width [6]. However, it has been suggested that ancient boat size would be smaller [7] and there has been long navigation history in small river in central Europe [8]. Therefore, 5m is taken as a standard to distinguish navigable river. Besides, to serve as the potential carrier of plague, the navigable river needs to be connected with other city. Enclosed water body with no navigable waterway to other city is also neglected. The closest distance of river is defined as the absolute distance from the coordinates to the nearest navigable water body. We then measure the width of the river at this nearest distance. In many cases this width does not represent the widest part of the river system. We assume that this variation would not affect much on our result. We only detect water body within a 10km radius area of our reference point. If no river is found within this periphery, 0m is entered as width of river and 10km is set as dummy for distance of river.

Spatial lag. Spatial lag is a measurement of the likelihood of whether plague outbreak at a certain unit is correlated with the previous outbreaks at the nearby units [9]. Existing research has proved that there were never any permanent plague reservoir in historical Europe [10]. In such words, any plague outbreak within our study period should be spread by a previously plague outbreak nearby. In analyzing spatial distribution of disease, failure to control such spatial clustering of outbreak events would lead to bias of correlation [11].

To illustrate how we calculate the spatial lag, we construct a hypothetical case as shown in Figure S2. This case hypothesized a plague outbreak case at city A. Within the study period, all cities B to G recorded at least one time of plague outbreak. However, we only looked into the plague record of the closest three points to city A, which are city B, city C and city D. The problem here is that the plague outbreak at city A might be transmitted from city G, looking into cases in only city B, C or D could not reflect it. However, in this study we are only aiming at controlling the effect of spatial clustering of plague events, not necessarily looking for the actual route of plague transmission in every plague outbreak. Therefore, we only consider the three closest plague outbreak points to city A. If there is sign of plague outbreak in city B, C or D in the previous two years, or the previous year, or the year when city A got its plague outbreak, one point is counted. As such, there should be a maximum of nine counts in these cases. It is further divided by the number of potential plague outbreak points nearby.

Coordinates. Coordinates reflect not only the spatial location of the plague outbreak. Longitude can reflect the potential inclination of distribution and latitude can show the possible effect of temperature to the distribution of plague outbreak. We use the coordinates as provided in the database of Büntgen, et al. [1] to construct the corresponding data.

Elevation. We pick elevation as another summary attribute of the impact of physical environment to plague outbreaks. Currently there is no study on the

linkage between elevation and plague outbreak. However, this indicator has been found influential to the spreading or clustering of other diseases [12,13]. The significance of elevation to disease clustering might be due to its negative association to population distribution [14]. In our model we will also control population density in order to cancel its correlation with elevation. For each plague outbreak point, we will measure its elevation with ArcGIS v10.1 by using the coordinates as provided by Büntgen, et al. [1].

Vegetation cover. There had been argument on whether plague spread more effectively in countryside than in urban areas [3]. To distinguish the degree of urbanization in Europe over time, we use the dataset of historical vegetation cover on usable land constructed by Kaplan, et al. [15] as our independent variable. The data is available for AD1000, AD1350, AD1400 and AD1850. For the missing year we estimate them by using linear interpolation.

Normalized population density. Population density is another attribute that can potentially reflect the degree of urbanization. For some other infectious diseases, the risk of infection can be associated with the population density of the potential host [16,17]. This metric we used in this part is derived by normalizing the population density with the percentage of usable land over time:

$$NPD^t = P^t / A * UL \quad \text{(Eq. 1)}$$

where NPD^t is the normalized population density at a given time; P^t is the total population of a country at a given time as provided by McEvedy and Jones [18]; A is the area of the country and UL is the percentage of usable land of the country as given by the work of Kaplan, et al. [15]. Assuming that human population only live in place suitable for cultivation, this index allows us to compare the population density of different countries on these usable lands over time. The dataset provided by McEvedy and Jones [18] is available for 50-year resolution. Missing information on population was filled up by assuming an exponential growth within the period.

Per capita GDP. It is possible that improvement in economic situation might represent the advancement in medical system and also the hygienic condition as a whole. On the other hand, economic fall down could be associated with famine and epidemic outbreak [19]. Per capita GDP data is extracted from the work of Maddison [20] and Bolt and Zanden [21]. Their dataset includes per capita GDP figures of historical Europe in the resolution of around approximately 50-year period. We construct the missing data by assuming linear growth of GDP within gaps. For regions with their first data-point found after AD1347, we assign value from the closest country nearby.

Basic laborer wage. Despite the inclusion of per capita GDP that reflects the economic situation in European countries over time, we also include the basic laborer wage that represents the lives of common workers back in historical Europe. The basic laborer wage serves as a surrogate for the life of lower class in Europe [22]. As described in the work of Allen [22], these wages were a benchmark of 'physiological minimum', which refers to the situation when the salary equals to

the minimum amount of diet that could be exchanged by money to survive. This figure probably indicates the most vulnerable group of people to diseases and the lowest quality of life at that time. The dataset of basic laborer wage is originated from the work of Allen [23]. The original dataset provides finest resolution to 1-year period in major European cities. We transform the dataset to country scale by combining data from the same countries together. Linear interpolation is performed for missing values in the temporal domain of the dataset. For region with their first data-point found after AD1347, value from the closest countries is assigned.

Consumer price index. In order to reflect the living standard and quality of life back in historical Europe, we use the consumer price index as a measure to the social well-being. It surrogates the general ability of purchase common goods and the conditional cost of maintaining the living standard over different spatial domains across time. The dataset is originated from Allen [23] in finest resolution of 1-year period. Estimation of data for intervening years was made by linear projection of data and also by borrowing figures from the closest data point available.

Table S1. Proportion of plague-infected cities against all cities at different distance interval to navigable rivers

Radius of measurement (R)	10km	7.5km	5km	4km	3km	2km	1km
Number of plague-infected cities within R	5309	5271	5216	5174	5065	4966	4461
Total number of cities within R[a]	200305	177590	142485	125139	99120	68145	37996
Percentage (%)	2.65	2.97	3.66	4.13	5.11	7.29	11.7

[a] Data of the total number of cities (N=918) were collected from the statistical information on European cities (Urban Audit) provided by Eurostat (http://ec.europa.eu/eurostat/statistics-explained/index.php/Statistics_on_European_cities); River data were collected from the stream line data (N=8543) in the HYDRO1k file, which was developed by the U.S. Geological Survey's (USGS) (http://earthexplorer.usgs.gov/).

Table S2. Summary statistics of the variables employed in our OLS estimates

Variable	Mean	Std. dev.	Min	Max	N
Number of count of plague outbreak	27.37	28.12	1	132	5559
Width of river (m)	84.63	99.21	0	868	5559
Distance of river from city centre (km)	1.17	2.27	0	10	5559
Spatial lag	0.26	0.41	0	3	5559
Distance from equator (degree)	47.95	3.90	36.36	58.37	5559
Longitude(degree)	4.21	5.97	-9.26	37.55	5559
Elevation (m)	153.43	174.28	2	1212	5559
Vegetation cover (%)	16.82	7.53	0.64	63.87	5559
Normalized population density (no. of person/km^2)	265.62	319.14	6.82	4187.45	5559
Per capita GDP (1990 Int. GK$)	1046.01	211.77	430.89	1753.18	5559
Basic wage (grams Ag/day)	4.76	1.36	0.57	9.11	5559
Consumer price index (Ag price)	0.90	0.41	0.13	4.298	5559

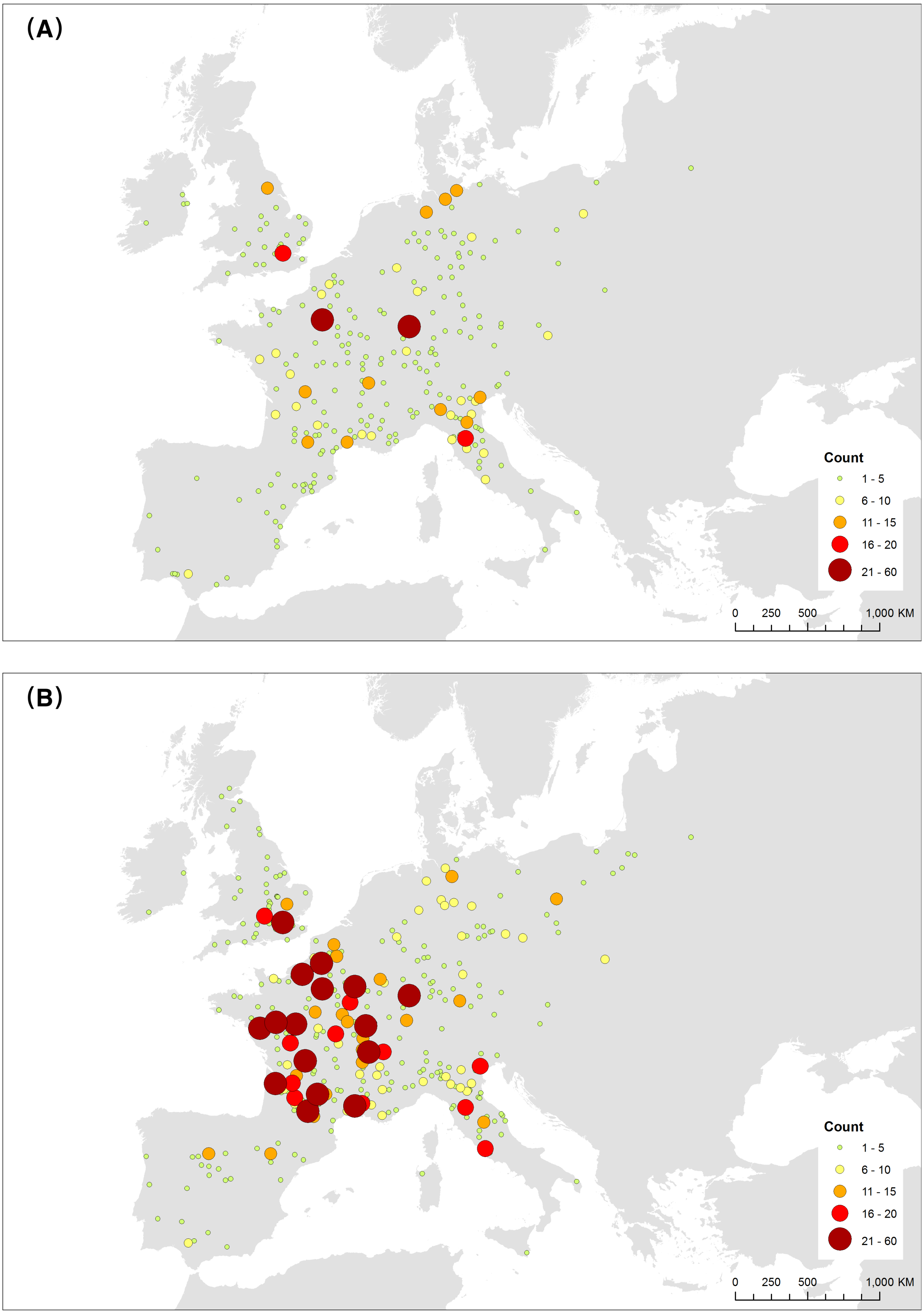
(A)
Count
1 - 5
6 - 10
11 - 15
16 - 20
21 - 60
0 250 500 1,000 KM
(B)
Count
1 - 5
6 - 10
11 - 15
16 - 20
21 - 60
0 250 500 1,000 KM

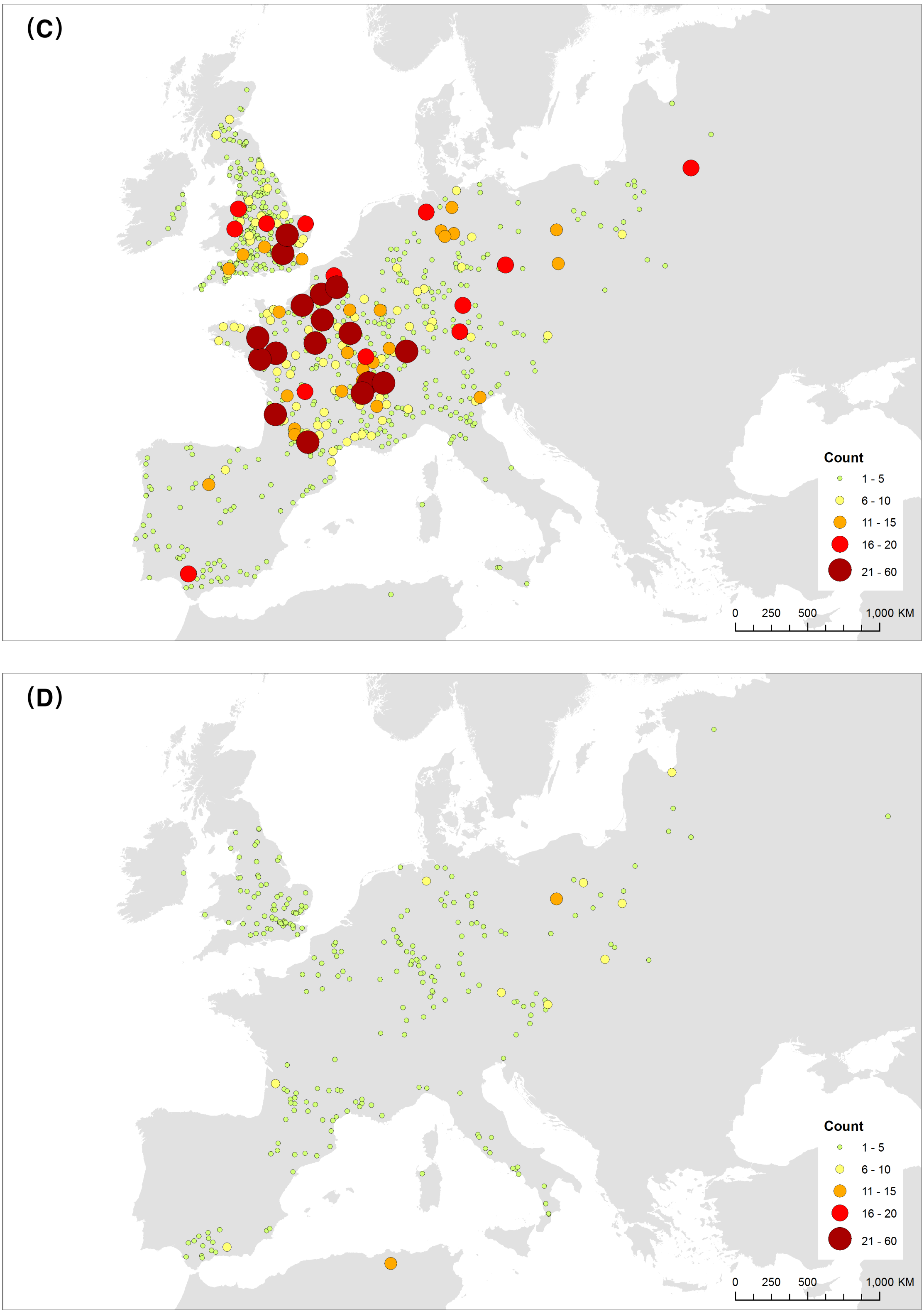

Figure S1. Spatial distribution of plague outbreak in Europe in different periods. The frequency of plague reoccurrence is corresponding to the colour spectrum. (A). AD1347 – 1449. (B). AD1450 – 1549. (C). AD1550 – 1649. (D). AD1650 – 1760. The maps are generated in ArcGIS version 10.1 (www.esri.com/software/arcgis).

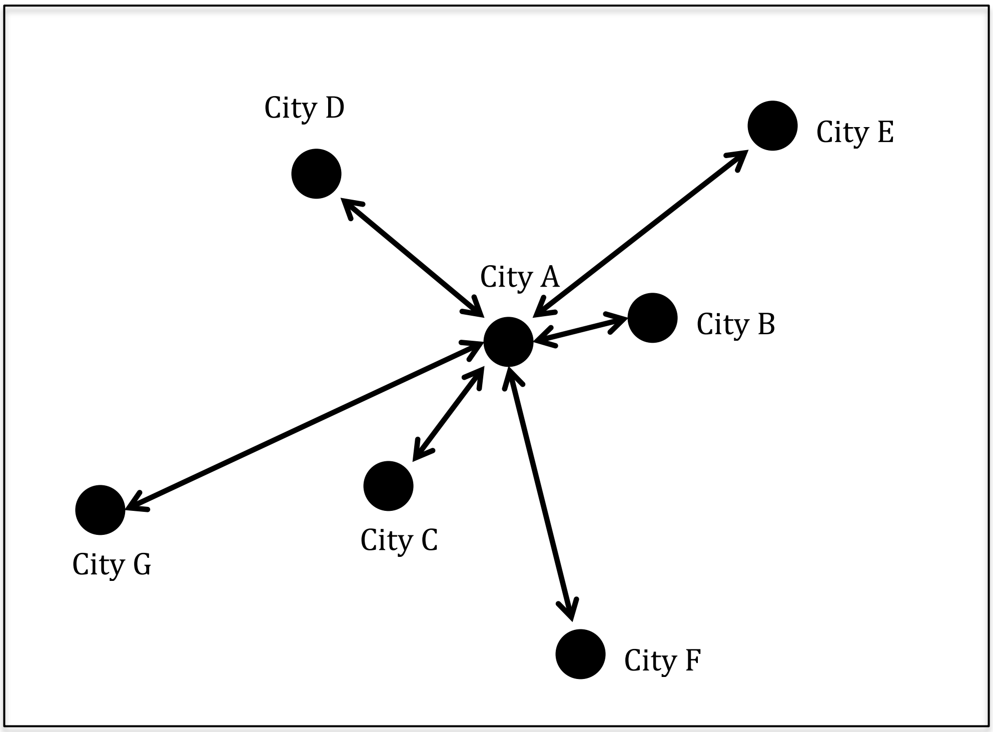

Figure S2. Graphic illustration to spatial lag

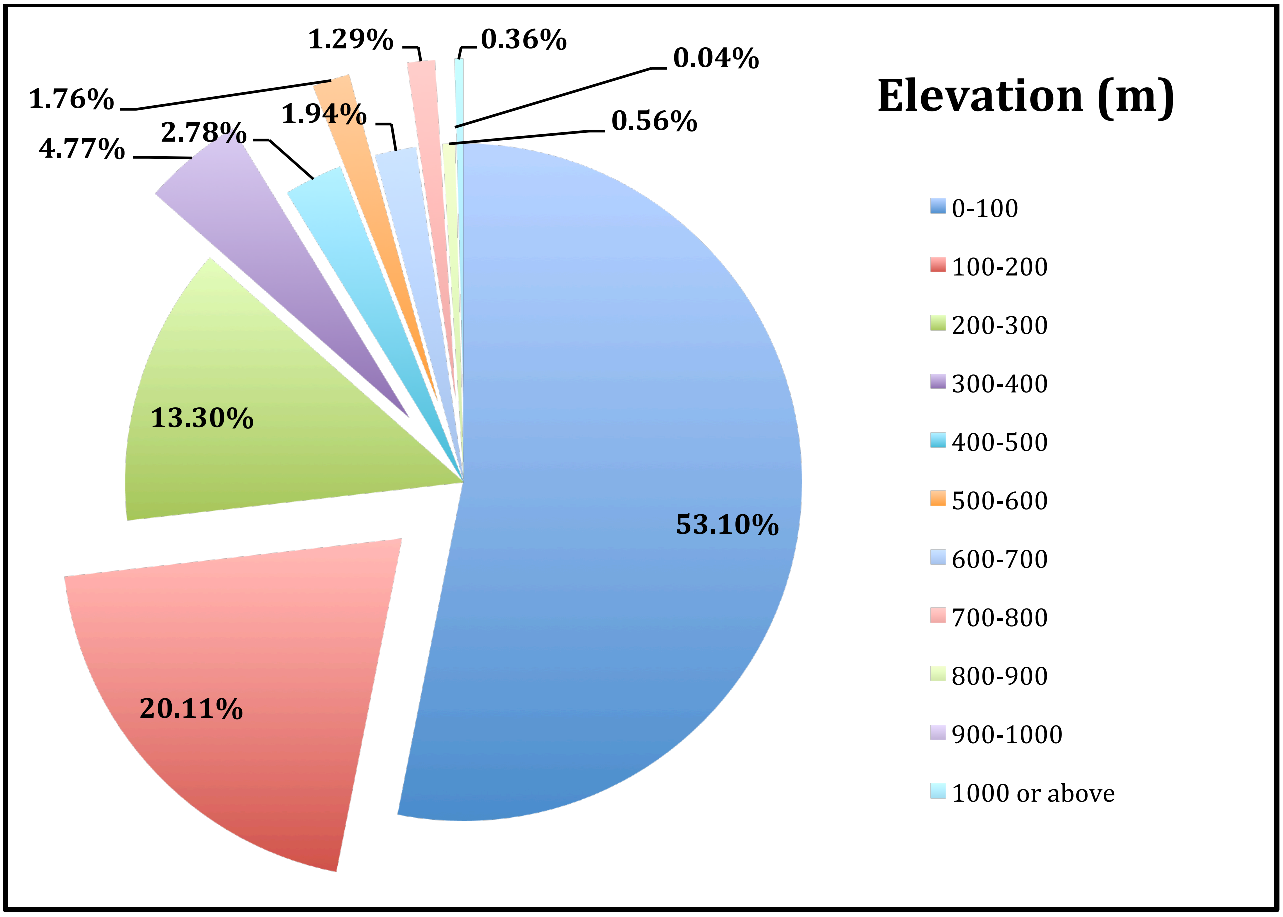

Figure S3. Distribution of elevation of plague outbreak incidences in Europe, AD1347 – 1760.

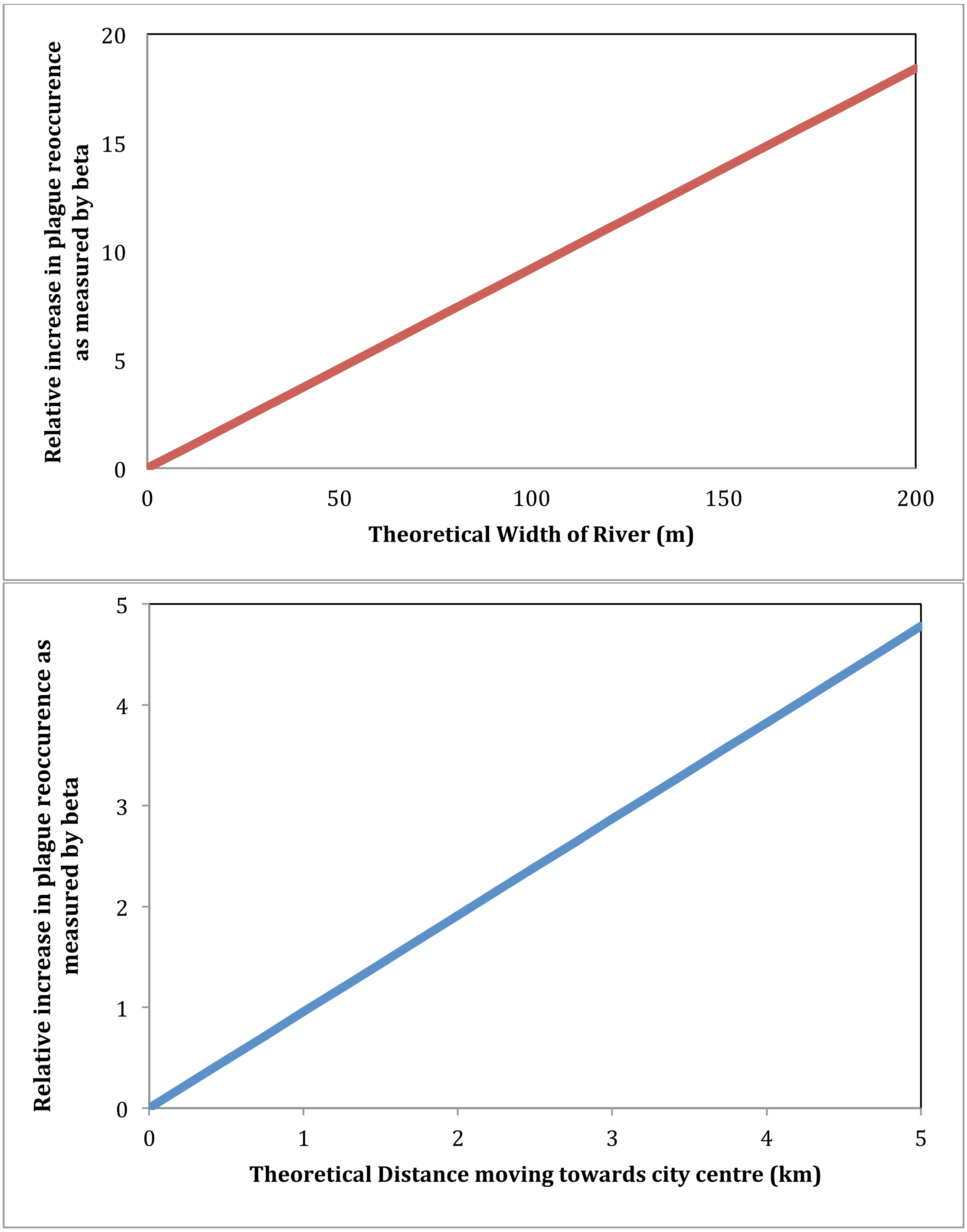

Figure S4. Theoretical impact of river to plague outbreak in Europe, AD1347 – 1760. (A). Theoretical width of river in relation to relative increase in plague reoccurrence. The standardized beta coefficient from column (5) of Table 2 is used. (B). Theoretical distance away from city centre in relation to relative increase in plague reoccurrence. The standardized beta coefficient was brought from column (5) of Table 2.

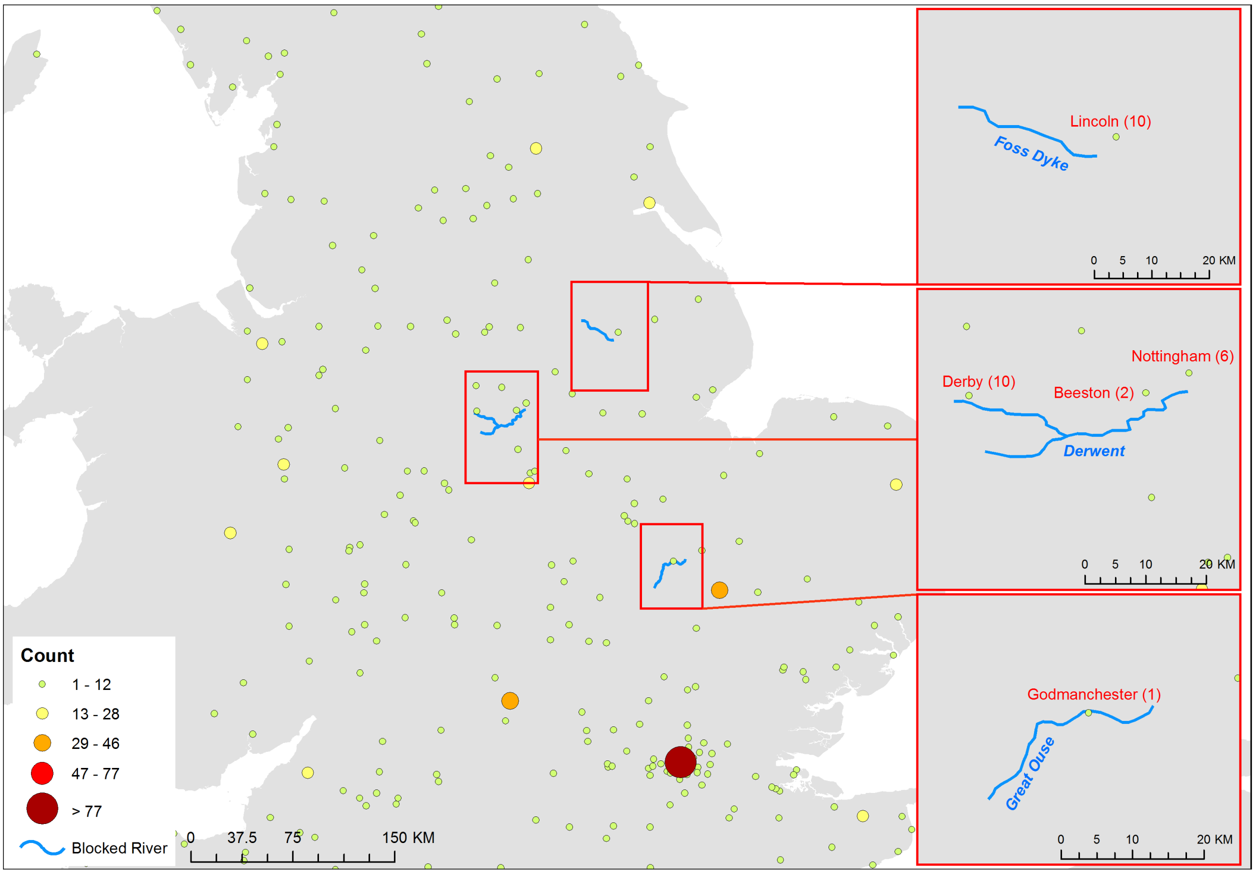

Figure S5. Maps showing Rivers Derwent, Greatriver Ouse and Fossdyke and the closest plague outbreak point nearby.

Explanatory note: In the case of River Derwent, it seems like plague could not spread easily to the blocking part of the river. At the two ends of blocking parts, Derby and Nottingham were recorded with ten times and six times of plague outbreak respectively. However, within the blocked canal of River Derwent, only Beeston was spotted with two times of plague outbreak. For the case of Fossdyke, no plague incidence was found at the upstream part after the blockage of the waterways. Lincoln, locating before the blocking part of the channel, recorded with ten times of plague outbreak during our study period. However, there is no record of its further spreading to the upstream and it may provide evidence that blockage of waterway stopped the spreading of plague. A similar situation would also be applied in Greatriver Ouse, no major outbreak would be found along the waterway except for one time outbreak at Godmanchester in 1605. However, the outbreak would be regarded as sporadic case. The maps are generated in ArcGIS version 10.1 (www.esri.com/software/arcgis).

References

1 Büntgen, U., Ginzler, C., Esper, J., Tegel, W. & McMichael, A. J. Digitizing historical plague. *Clinical infectious diseases* **55**, 1586-1588 (2012).

2 Tracy, J. D. *The Rise of Merchant Empires: Long Distance Trade in the Early Modern World 1350-1750*. Vol. 1 (Cambridge University Press, 1993).

3 Benedictow, O. J. *The Black Death, 1346-1353: the complete history*. (Boydell & Brewer, 2004).

4 Ashton, T. S. *The industrial revolution 1760-1830*. Vol. 109 (In the Hands of a Child, 1966).

5 Biraben, J.-N. *Les hommes et la peste en France et dans les pays européens et méditerranéens*. Vol. 2 (Mouton Paris, 1975).

6 PIANC. *Standardization of inland waterways' dimensions*. (General Secretariat of PIANC, 1990).

7 McGrail, S. Early Ships and Seafaring. European Water Transport. *Barnsley: Pen & Sword Archaeology* (2014).

8 Eckoldt, M. Navigation on small rivers in Central Europe in Roman and Medieval times. *International Journal of Nautical Archaeology* **13**, 3-10 (1984).

9 Buhaug, H. & Rød, J. K. Local determinants of African civil wars, 1970–2001. *Political Geography* **25**, 315-335 (2006).

10 Schmid, B. V. *et al.* Climate-driven introduction of the Black Death and successive plague reintroductions into Europe. *Proceedings of the National Academy of Sciences* **112**, 3020-3025 (2015).

11 O'Loughlin, J. *et al.* Climate variability and conflict risk in East Africa, 1990–2009. *Proceedings of the National Academy of Sciences* **109**, 18344-18349 (2012).

12 Bødker, R. *et al.* Relationship between altitude and intensity of malaria transmission in the Usambara Mountains, Tanzania. *Journal of Medical Entomology* **40**, 706-717 (2003).

13 de Magny, G. C. *et al.* Environmental signatures associated with cholera epidemics. *Proceedings of the National Academy of Sciences* **105**, 17676-17681 (2008).

14 Cohen, J. E. & Small, C. Hypsographic demography: The distribution of human population by altitude. *Proceedings of the National Academy of Sciences* **95**, 14009-14014 (1998).

15 Kaplan, J. O., Krumhardt, K. M. & Zimmermann, N. The prehistoric and preindustrial deforestation of Europe. *Quaternary Science Reviews* **28**, 3016-3034 (2009).

16 Lafferty, K. D. & Holt, R. D. How should environmental stress affect the population dynamics of disease? *Ecology Letters* **6**, 654-664 (2003).

17 Schmidt, W.-P. *et al.* Population density, water supply, and the risk of dengue fever in Vietnam: cohort study and spatial analysis. *PLoS Med* **8**, e1001082 (2011).

18 McEvedy, C. & Jones, R. *Atlas of world population history*. (Penguin Books Ltd, Harmondsworth, Middlesex, England., 1978).

19 Zhang, D. D. *et al.* The causality analysis of climate change and large-scale human crisis. *Proceedings of the National Academy of Sciences* **108**, 17296-17301 (2011).

20 Maddison, A. *The world economy volume 1: A millennial perspective volume 2: Historical statistics*. (Academic Foundation, 2007).
21 Bolt, J. & Zanden, J. L. The Maddison Project: collaborative research on historical national accounts. *The Economic History Review* **67**, 627-651 (2014).
22 Allen, R. C. in *XIV International Economic History Congress, Helsinki.*
23 Allen, R. Allen-Unger Database: European Commodity Prices 1260-1914. *On-line:* http://www2*. history. ubc. ca/unger/htm files/new grain. htm* (2007).
